# Transitioning practices of vegetable small-scale actors in Vietnam: an interplay of food safety, labor demand, and soil environment

**DOI:** 10.1007/s10460-024-10636-6

**Published:** 2024-10-23

**Authors:** Quoc Nguyen-Minh, Raffaele Vignola, Inge D. Brouwer, Peter Oosterveer

**Affiliations:** 1 Environmental Policy Group, Wageningen University, Postbus 8130, 6700 EW Wageningen, Netherlands; 2 Water Systems and Global Change Group, Wageningen University, P.O Box 47, 6700 AA Wageningen, Netherlands; 3 Division of Human Nutrition, Wageningen University, P.O. Box 17, 6700 AA Wageningen, Netherlands

**Keywords:** Food systems, Food safety, Practice theory, Food system transitions, Smallholders, Vegetables

## Abstract

Food safety is a critical and persistent issue that challenges the sustainability of agri-food systems in Vietnam. The government has launched multiple food safety initiatives, but there is limited understanding of their contribution to changing the practices of small-scale producers and distributors. This study explores these changing practices by applying Social Practice Theory (SPT) to analyze the transitions in everyday routines of small-scale vegetable producers while being embedded in socio-institutional contexts of agri-food system transitions. We conducted semi-structured interviews and survey with small-scale food producers and distributors in Hanoi, Vietnam to examine the transitions in production and post-production practices over the last 20 years and the intersection between smallholding practices and cross-level dynamics. The study revealed, contrary to some common perceptions, that smallholder producers are transitioning towards food safety, with the use of more bio-pesticides and eco-friendly pest control methods. The smallholders also reproduce a variety of (sustainable) intensification practices, including crop rotation, organic fertilization, and soil cultivation, to sustain soil fertility and save labor. However, there are no clear patterns of change for post-production practices, although they have been diversifying under the impacts of urbanization. The findings highlight the interplay of food safety, labor, and soil environment in shaping the transitions of smallholder practices. We suggest that success in improving safety in production practices is feasible, but that this requires more thorough interventions in distribution and consumption practices to transform the food systems at large.

**Supplementary Information:**

The online version contains supplementary material available at 10.1007/s10460-024-10636-6.

## Introduction

Food safety concerns remain high on the agenda in many developing countries. They are amplified and complicated by the dynamics associated with the development process, including income growth, rapid urbanization, global trade integration, technological progress, and changing consumer demands and lifestyles (Bishwajit 2014). People in lower- and middle-income countries (LMICs) are generally more vulnerable to foodborne diseases due to higher exposure and sensitivity to chemical and biological hazards (Ortega and Tschirley 2017). However, the design and implementation of food safety regulations in LMICs face critical challenges due to the often ‘heterogeneous and fragmented’ food value chains, which mostly interact through informal institutions (Grace 2015). Thus, the promotion of formal food safety rules, including international and domestic standards, faces high transaction costs and risks, excluding many small-scale actors from the food supply chains (Mercado et al. 2018). Researchers and policy-makers inquire how smallholders can adopt food safety practices and successfully adapt to changing market requirements (Unnevehr 2015).

As small-scale producers and distributors^1^ often constitute the dominant and incumbent parts of the food systems in the developing world, small-scale practices are crucial not only for food safety but also for food security, nutrition, and the environment more generally (Gomez 2020). Alterations in the everyday practices of small-scale actors can contribute to incremental changes that eventually steer the sustainability transition of food systems (Bui et al. 2016; Huttunen and Oosterveer 2017). They can shape the pathways of food system transitions, either by embracing large-scale, standardized, and modern production (Gómez and Ricketts 2013; McCullough et al. 2008) or by incorporating alternative, localized, and ecosystem-based practices (Corsi et al. 2018; Watts et al. 2005). However, understanding small-scale practices is never simple since smallholders are not a homogeneous group. Different production factor endowments (land, capital, and labor), various policy interventions and institutions, and varied levels of technological and market access can all influence the manifold practices of small-scale actors, contributing to the diverse roles that smallholders play in agri-food transitions (Burkitbayeva and Swinnen 2018).

Considering the essential and dynamic role of smallholders in addressing food safety issues, our study focuses on small-scale actors in the vegetable supply in Vietnam. Since the economic reforms in the late 1980s, vegetable production and consumption have been promoted to improve diet quality and enhance smallholders’ incomes. Nevertheless, concerns about the safety of vegetables remain prominent due to the regularly reported poor pesticide use practices (Hoi et al. 2009). Multiple interventions have been launched by the government or non-government organizations (NGOs) to promote practice changes towards safer vegetable supply, yet there is limited understanding of whether such changes have actually taken place among small-scale actors and been influenced by such interventions.

The main aim of this study is to understand the transitioning practices of small-scale producers and distributors in Vietnamese vegetable systems. We analyze how the practices of Vietnamese vegetable smallholders have changed over the last 20 years and what intervention points can promote transitions toward safe practices. The first question is addressed by using the lens of social practice theory (SPT), while the second is answered by combining the findings from SPT analysis with the concepts and insights from the sociotechnical transitions literature. The study can contribute to the growing body of studies integrating practice theory with food system transition research (El Bilali 2018). Empirically, the focus on Vietnam can shed light on how smallholders in a post-socialist emerging economy have adapted their practices in the context of globalization and modernization and what the prospects are for better engaging them as pivotal actors in the transition toward achieving the global Sustainable Development Goals (SDGs).

## Theoretical background

Social practice theory (SPT) refers to a series of theories developed around the concept of ‘practice’, with the aim of overcoming the divide between individual human agency and social structure in social sciences (Spaargaren 2011). From this perspective, daily human actions do not suddenly arise from immediate rational decision-making processes but mostly follow a routinized, habitual, and taken-for-granted manner. Practice-based approaches, therefore, consider such routinized behaviors (practices) as the main unit of analysis instead of individuals who are considered the carriers of such practices (Reckwitz 2002).

Practices are made up of several interconnected elements, comprising mental, bodily, knowledge, and material dimensions (Reckwitz 2002). Schatzki (2002) argued that practice is a ‘nexus of doings and sayings’, meaning the activities and their discursive expression ‘hang together’ by rules and norms, know-how, and ‘teleo-affective structures’ (i.e., the ends, goals, projects, values, beliefs, and emotions that give a direction to practices). Such norms and teleo-affectivities are needed for the organization and ordering of practice elements, so they resemble other terms such as ‘organizing principles’ (Giddens 1984) or ‘conscious logics’ of a practice (Huttunen and Oosterveer 2017). Schatzki also distinguished between practice-as-entity (the idealized form of practices that are collectively recognized by people over time) and practice-as-performance (various enactments of practices in specific temporal and spatial circumstances) (Warde 2005). Practices can be simple (dispersed) or complex (integrative) or can be bundled together when sharing some mutual elements (Brons and Oosterveer 2017).

In our study, ‘small-scale practices’ are (bundles of) practices that small-scale vegetable production and distribution actors enact and reproduce. They include cultivation, seeding, fertilizing, crop protection, harvesting, post-harvest soil management (production), and preprocessing, transportation, wholesaling, and retailing (distribution). Bundles of practice that share the same meanings, such as ‘food safety practices’, or the same organizing approach, such as ‘cooperative practices’, can also be identified. The study intends to comprehend the overall dynamics of small-scale practices (as entities), while the variations in practice performances help illustrate and enrich comprehension.

The study focuses on the long-term dynamics of small-scale practices, i.e. their transitions. Transitions can be understood as changes from one state to another (Geels and Schot 2007), hence practice transitions refer to the shifts from old, established practices to new ones. To study practice transitions, we apply the widely adopted approach by Shove et al. (2012) to analyze changes in three practice elements: *meanings* (symbols, norms, values, beliefs, or principles that govern actions), *materials* (physical environment, infrastructures, technologies, inputs, and tools), and *competences* (skills and know-how). These elements are independent in some way, but their coexistence enables linkages to be made and practices to be reproduced. Changes in smallholder practices will occur whenever linkages are broken and recreated owing to the introduction of new elements or the impacts of changing sociopolitical contexts.

In addition to zooming in on the practice changes, we also zoom out to explore what factors facilitate or hinder the processes of transitions. The idea of zooming out means ‘switching theoretical lens’ and investigating the possible connections among practices across space and time (Nicolini 2009). The (horizontal) connections among practices might be collaboration or competition based on how they share, reproduce and distribute elements (Shove et al. 2012). Yet from a vertical viewpoint, small-scale practices and their elements are embedded in the broader arrangements of multiple institutions and practices that constitute a selection environment for blocking or promoting certain practices.

To analyze vertical connections, we combine practice analysis with transition theory, especially the Multi-Level Perspective (MLP) which focuses on the co-evolution of the three levels of niches, regime, and landscapes that bring about the transition (long-term, fundamental changes) of socio-technical systems. The *regime*(s) are the dominant part of the system, comprising coherent, stable and functionally important networks of practices and arrangements, while *niches* are smaller and less stable but have the potential to induce transformative changes (Holtz et al. 2008; Smith and Raven 2012). In the context of food systems in Vietnam, the regime refers to the dominant smallholder-led production and distribution networks, while niches are agri-food initiatives or innovations. There are, however, no clear boundaries between niches and regimes, since niches can grow and influence regime restructuration or replace the regime (Grin et al. 2010). Meanwhile, both niches and regimes are separate from, yet embedded in, *landscapes*, which are broader factors beyond the direct control of niche/regime levels. The MLP and SPT share similar concerns and assumptions on the tension between change and stability and provide different yet complementary lenses for conceptualizing complex dynamics at micro-level everyday routines and macro-level food systems (Hinrichs 2014).

**Fig. 1:**
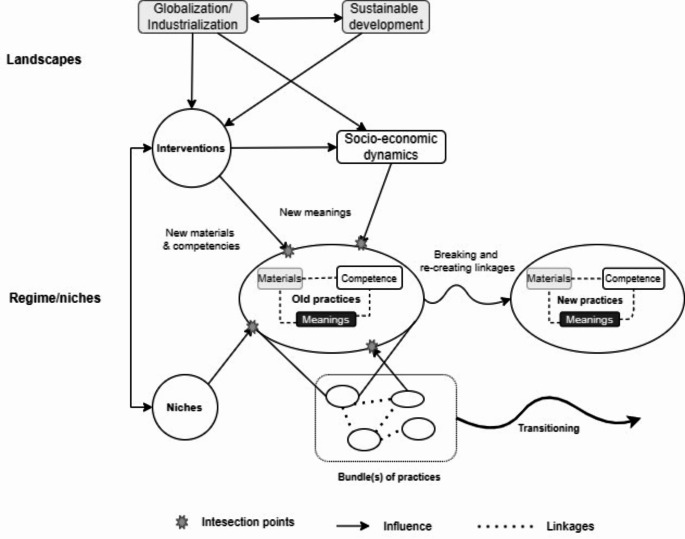
Analytical framework. Source: Own elaboration, primarily based on Shove et al. (2012) and Hargreaves et al. (2013).

The analytical framework is shown in Fig. 1. In applying the analytical framework, we first identify the key practice changes, before examining linkages between practice elements (meanings, materials, competences) aligned with the changes (zooming in). Next, we consider the interactions between practices, elements and broader socio-technical dynamics, including the institutional changes of Vietnamese agri-food systems and other socio-economic processes (zooming out). Those national-level dynamics are influenced by global landscape changes, for which we use the argument outlined by Spaargaren et al. (2012) that two global movements– globalization and sustainable development– are the two main ‘organizing principles’ (or ‘global attractors’) that guide the transition dynamics of food practices. Lastly, we identified the intersection points between the cross-level dynamics and practices, which were conceptualized as ‘*friction points’* (Hargreaves et al. 2013) or ‘*fractures’* (O’Neill et al. 2019), which can hinder or open opportunities for innovations in practice and therefore be the target of interventions for policy-makers and practitioners.

### Background on Vietnamese vegetable systems

The vegetable production system in Vietnam is dominated by small-scale producers, primarily located in the Red River Delta (RRD), Mekong River Delta (MRD), and Lam Dong Province. Vegetable producers typically have landholdings of approximately 0.25 ha in the North and 0.5 ha in the South, which is among the lowest in the developing world (Rapsomanikis 2015; van Wijk 2010). The prevalence of smallholders in vegetable production is the result of a transition process since the early 1990s when the collapse of cooperative systems gave rise to the rapid emergence of small-scale family farms with equitably small land tenures (owing to the 1993 land distribution policy) (Do and Iyer 2008). Vegetables are important cash crops that bring additional income for farmers beyond traditional rice cropping, accounting for 83% of income from crop production in rural areas and 89% in the peri-urban areas of the RRD (Huong et al. 2013a).

These small-scale producers are strongly connected to traditional distribution networks, which comprise collectors, wholesalers, wet market retailers, and street vendors (Figuié and Moustier 2009). Vegetable distribution networks expanded in the 1990s, when many rural and peri-urban farmers, mostly female, migrated and worked as street vendors or wet market stallholders in cities (Moustier et al. 2008). Since the 2000s, small-scale retailers have faced increasing competition from emerging supermarket chains (Maruyama and Trung 2012).

Food safety has been a long-lasting issue in Vietnamese vegetable systems. Economic pressure and the liberalization of agricultural input markets have led to the increased use of agrochemicals. Cases of pesticide overuse, including high-toxicity types, have been reported over the years (Hoi et al. 2009; Schreinemachers et al. 2020). Given the growing public concerns about food safety risks, the government and NGOs have launched several initiatives to enhance food safety control and promote safer practices among smallholders. Some of the initiatives involve the establishment of ‘safe vegetable production’ zones (*rau an toàn*, abbrev. RAT), the strict prohibition of high-toxicity pesticides (Hoi et al. 2016), and the promotion of safety guidelines like Integrated Pest Management (IPM) and certificates like Vietnamese Good Agricultural Practices (VietGAP) (Ngo et al. 2019; Pham 2017). The government also endorsed modern retail outlets owing to the notion that they can be more effective in food safety control (Wertheim-Heck et al. 2015).

While having varying successes at local scales, these policies still do not sufficiently address the food safety problem. Some studies attribute the persistence of food safety issues to institutional weaknesses (limited state capacity to enforce food safety control, lack of public‒private participation and coordination) (Pham and Dao 2016; Van Hoi et al. 2013) or the lack of economic incentives to adopt safety standards (Hoang 2020). Wertheim-Heck et al. (2014, 2015) used practice theory to explain how wet market shopping is an important part of the daily routine of consumers, and food safety strategies (mostly based on trust relationships with retailers) are embedded in such routinized practices. Wertheim-Heck questioned how the retail modernization strategy ignores the diversity of consumer practices and lacks the flexibility to include different ‘real world’ consumption practices. However, little is known about how effectively such policies and initiatives have shaped the reproduction of safe and sustainable practices among small-scale producers. A common critique is that the government approach is too productivity-focused with little acknowledgement of market linkages and limited outreach to poor, marginalized producers (Friederichsen et al. 2013; Linh 2001).

## Methodology

**Fig. 2:**
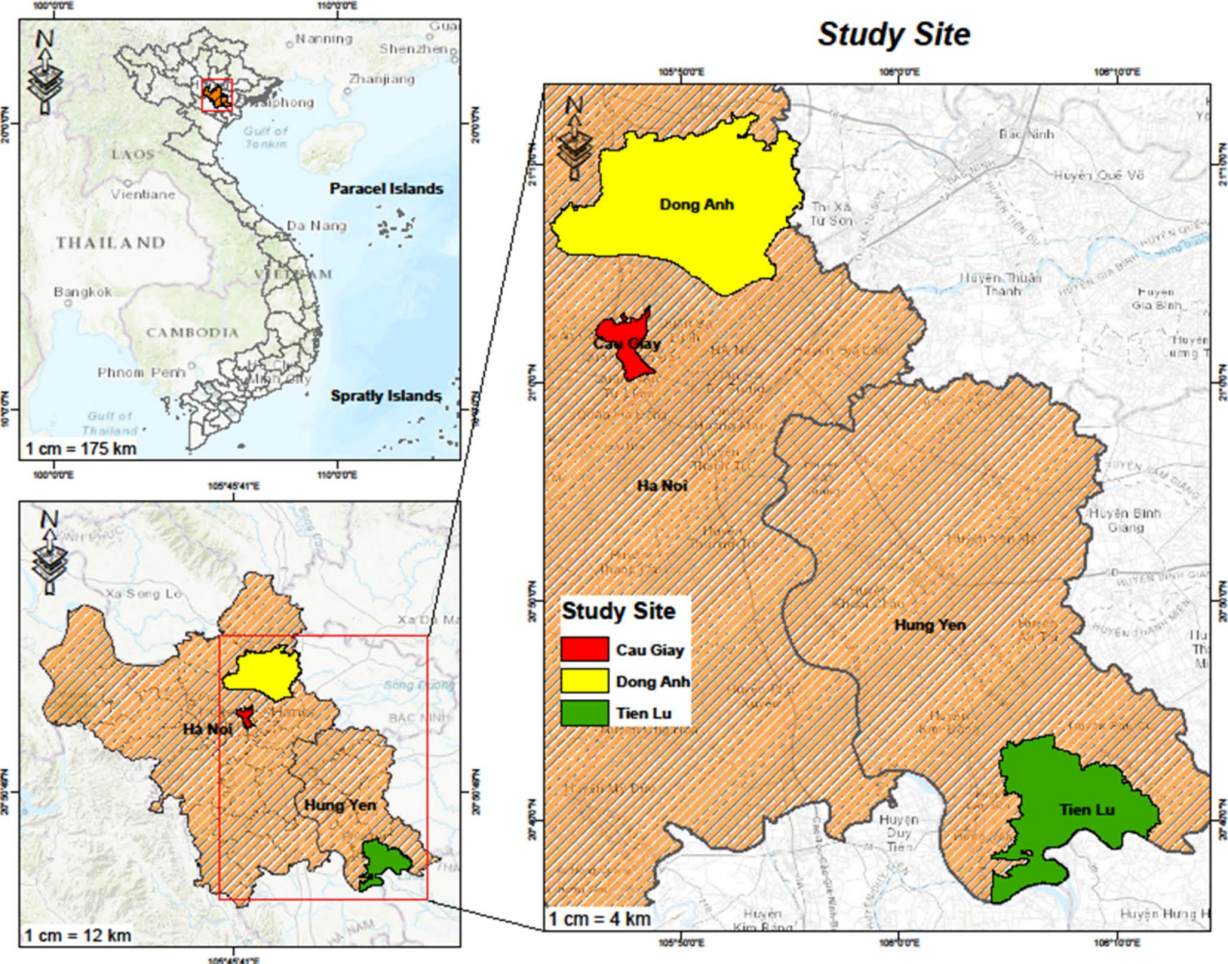
Location of the study sites. Source: own work with the use of ArcGIS Pro 2.8

### Data collection

The data collection was carried out in several steps. First, we conducted a desk study and key informant interviews to gain an overview of food-related regulations and institutions and sociotechnical innovations (niches) in Vietnamese vegetable supply chains. The desk study utilized literature from various sources including scientific articles, government and NGO reports, and legislative documents, while the key informant interviews involved experts and practitioners with more than 10 years of experience in vegetable supply and agri-food controls (see *Supplemental material*). The outcomes from this step provided a basis for the study’s emphasis on small-scale supply practices (often seen as the key driver of food safety issues). It helped outline different categories (bundles) of production and distribution practices, informing the design of research questions in the next step.

Second, on-site small-scale vegetable practices were primarily studied using semi-structured interviews, a survey and participant observation. The fieldwork took place between October 2020 and February 2021 in three districts, Cau Giay, Dong Anh (Hanoi), and Tien Lu (Hung Yen), all in the RRD, Vietnam, representing urban, peri-urban, and rural areas, respectively (Fig. 2). The selection of study sites within the RRD is based on the consideration of close intra-regional linkages between vegetable production and consumption activities, as well as the diversity of food environments along the urban-rural transect (Huynh et al. 2021). Dong Anh is a RAT zone designated by the Hanoi municipal government, supplying vegetables to the urban core of Hanoi. Meanwhile, Tien Lu and Cau Giay represents the rural and urban districts with strikingly different food environments, with the former having only informal wet markets with little development of modern retailing, and the latter having multiple retail formats—including modern channels, traditional wet markets, and street vending—coexisting and thriving. We selected these sites with the assistance of local authorities and local research networks. In each district, we chose two communes or markets where typical vegetable production and distribution practices were taking place.

We used purposive snowball sampling to select producers, paying attention to the diversity of vegetable farmers (with or without cooperatives, certified or non-certified), while wholesalers, wet market retailers, and street vendors were selected using convenience sampling at traditional markets. A total of thirteen producers and seven distributors were interviewed, while a further 56 producers and 67 distributors were surveyed. Overall, producers in both the survey and interviews have shown to have small and fragmented landholdings (see the general characteristics of the survey respondents in *Supplemental material*). Multiple respondents are engaged in both production and distribution practices, including collecting, pre-processing, and retailing, especially those joining cooperatives or retailing at peri-urban and rural markets.

The qualitative interviews, which lasted between 45 and 90 min, consisted of questions about how specific practices have been reproduced and changed, and how those changes are linked to different key meanings, materials, and competences. To trace back the historical development of practices, we used questions such as “How did you exercise this practice in the past 5–20 years?” and “When and why have the changes happened?”. We did not attempt to build general timelines of livelihood trajectories and life courses but focused on identifying instances of key practice changes and the settings that enable them. Meanwhile, the survey comprises questions that inquire respondents about their main bundles of practices, the current situation, and their self-awareness of practice dynamics. The interviews and survey were combined with observations, as the main researcher conducted farm and market visits on several days in October 2020 and February 2021 to observe local production and retailing practices.

Finally, it is important to note that the study is qualitative and explorary in nature (Ahmed et al. 2007). The main findings were drawn from the qualitative interviews, meanwhile the survey data were instrumental in corroborating the interview findings. The use of different methods helps enhance the interval validity of the research (Meijer et al. 2002). However, due to the small sample size and the use of non-probability sampling methods like convenience and snowball sampling, the survey results are limited in external validity and cannot be used to generalise the entire dynamics of small-scale actors in the RRD vegetable systems (Robinson 2014).

### Data analysis

The interviews were recorded, transcribed and managed by Atlast.ti, while survey data were extracted into Excel and analyzed (descriptively) using SPSS. We coded the interview transcripts inductively and deductively following the analytical framework (Fig. 1). After the initial descriptive coding, the codes were categorized using the identified practice bundles and the key associated elements, and new codes and categories were added in the second coding round. We identified major themes that characterize the transitions of different bundles (zooming in). We further constructed narratives on such practice transitions and connected them with dynamics across levels, especially arrangements regarding food safety (zooming out). Results from the interviews and survey were also selectively reported to back the themes and narratives. They would be separately denoted as ‘interview results/findings’ and ‘survey results’ to avoid confusion.

## Results: ‘zooming in’ on small-scale vegetable practices in transition

### Production practices

The study identified several transitioning practices among vegetable producers, whereby two bundles of practices stand out, namely, ‘crop protection’ and ‘(sustainable) intensification practices’ (Table 1). The first bundle is organized around the common purpose of preventing pests, weeds, and pathogens, while the second is aimed at enhancing productivity and yield. Some practices have disappeared, as they are considered too effort-consuming and not cost-effective and have been replaced by others (for example, “seed preservation practices” owing to the possibility of purchasing high-yielding seeds in the market).

**Table 1:** Vegetable production practices in transition and their associated meanings, materials, and competence Source: own elaboration based on the interviews

Production practices	Meanings	Materials	Competence
Crop protection practices			
Using biological pesticides	- Enhance safety and reduce health risks for both producers and consumers - Reduce pre-harvest intervals (PHI) - Environmental concerns - Ensure ecological integrity - Reduce pesticide resistance - Reputation of the cooperatives - Social responsibility	- Pesticide labeling - Pesticide shops with sellers able to provide advice - Policies and state capacities to prohibit high-toxicity chemical pesticides - Higher pesticide costs - Pre-harvest intervals - Certification (if possible)	- Skills and knowledge about the impacts of agrochemicals and safer pesticide usage (from IPM training) - Knowledge about pesticide labeling and the benefits of biological pesticides - Ability to supervise (cooperatives)
Using both chemical and biological pesticides	- Productivism: cost-effectiveness of chemical pesticides - Balance effectiveness and risks	- Pesticide labeling - Pesticide shops - Personal protective equipment (PPE) - Pre-harvest intervals	- Ability to read pesticide labels and follow instructions - Ability to observe plant health conditions - Learning and experimenting
Using ‘homemade pesticides’ and physical methods	- Minimize safety and health risks (especially for self-subsistence) - Take advantage of cheap and locally available ingredients (lemon, chili…) - Ensure ecological integrity	- More work and dedication - Natural insect repellents (lemon, chili, garlic.) - Tools (insect traps and nets) - Certification (if possible)	- Skills and knowledge about ecosystems and agroecological methods (from IPM training) - Ability to observe plant health conditions - Learning and experimenting
Waste management	- Reputation of the cooperatives - Social responsibility - Environmental concerns	- Waste containers in the field - Waste management plan and workers - Facilities for transporting, disposal, and treatment of pesticide packaging	- Knowledge about the impacts of pesticide packaging waste - Ability to supervise (cooperatives)
(Sustainable) intensification practices			
Using mostly organic fertilizer (esp. chicken manure)	- Maintain soil porosity and fertility - Reduce risks of pests and diseases - Utilize manure from one’s farm - Enhance productivity and incomes	- Available manure from chicken farms - Transportation and storage facilities - Area for composting manure - Mineral and synthetic fertilizers (if needed) - Available time - Soil properties	- Know-how from extension programs as well as traditional knowledge - Ability to observe plant and soil conditions - Ability to optimize the usage of organic fertilizers according to plant conditions - Learning and experimenting
Using mostly synthetic fertilizers	- Enhance productivity and incomes - Ensure the essential nutrients for vegetables - Reduce workload and time	- Mineral and synthetic fertilizers - Finance - Manure (if possible)	- Ability to optimize the usage of mineral/synthetic fertilizers - Learning and experimenting
Post-harvest soil management (incl. composting)	- Improve soil porosity - Remove habitats for pests and pathogens - Ensure soil quality for maintaining yields - Recycle nutrients from agricultural residues	- Work and time - Pumping machine and water - Limestone powder - Available area for gathering and composting residues - Weather (sunlight) - Soil properties	- Cultivation technique - Time management skills - Ability to observe soil conditions - Learning and experimenting - Know-how from IPM training, extension program, and traditional knowledge
Crop rotation	- Improve soil fertility and nutrients - Recover and maintain yields - Limit pests and diseases - Seasonal suitability	- Work and time - Compulsory protocols of soil preparation - Weather - Soil properties	- Ability to observe soil conditions - (Traditional) knowledge about seasonal crops and farming calendar - Learning and experimenting
Using lightweight machinery	- Balance between labor saving and environmental impacts - Reduce workload (due to loss of labor)	- Lightweight machinery (plowing machine, cultivator, land mower) - Renting cost - Workload	- Cultivation technique - Skills to use and maintain machinery
Using cattle and manual labor	- Concerns for soil health and porosity - Make use of available labor	- Workload - Cattle and buffaloes - Tools (cultivator…)	- Cultivation technique - Ability to manage cattle and buffaloes

### Crop protection practices: transition toward food safety

There has been a switch from chemical to biological pesticides, as stated by most interviewed producers. The survey results also showed that 90% are now using bio-pesticides, while 27.5% applied physical measures and natural repellents. Only five survey respondents admitted to using chemical pesticides, mostly in the early phase (4–5 days) of vegetable maturity.

Besides, the interviewees confirmed their compliance with the pre-harvest intervals (PHIs), which specify a certain waiting time for the vegetables to be harvested after the last application of pesticides. PHIs can be found on the labels, and biopesticides usually require a shorter time (3–7 days). This implies that the notion and protocols of food safety have been widely diffused among vegetable producers in Dong Anh. This signifies a transition in pest management practices from the unrestrained uses of high-toxicity pesticides (Hoi et al. 2009, 2016) to more regulated, informed, and safer usage.

The use of pesticides and other preventive methods are the central practices in the bundle. One major activity is buying pesticides from dealers. When coming to the shops, many smallholders now reproduce the practice of *selecting and purchasing pesticides with ‘biological’ labels*. Biological pesticides mostly only target pests and have little to no harmful effects on water, soil, and ecosystems (Radcliffe et al. 2008). As biopesticides are more selective in treating specific types of pests, the role of sellers becomes more central. The interviews reveal that the producers hardly remember the names and functions of the products; hence, they often have to rely on trust relationships with the pesticide dealer and receive their advice on suitable pesticides and herbicides. “There are too many types to choose, and I did not learn high to know their contents, so it is better to ask the dealer” (vegetable producer, #40).

Although biopesticides were introduced more than 20 years ago, their usage has only gained popularity in the last 5–10 years according to the interviews. The increasing availability of biopesticide products, with labelling and distribution channels, was the key element for this transition. Another factor is the role of the local Plant Protection Department (PPD) since they are responsible for enforcing the national regulations to prohibit the sale and use of high-toxicity chemicals and promote safer products of biological ingredients.

“Chemical pesticides are cheaper than biological ones […] The vegetables that we spray chemical pesticides on grow to be better-looking than those with biopesticides. But people in my village now just don’t use chemicals anymore. Even if we want to use them, the shops are no longer selling them since the staff from the district and the city (Hanoi) regularly visit the shops and prohibit their sale.” (a vegetable producer in Dong Anh, #12).

Biopesticide users primarily attribute concerns for safety, health, and the environment as the central meanings. Being directly exposed to pesticides, farmers are worried about their own health, as well as about the dispersal of those chemicals into the environment. Another driver is their sense of social responsibility and reputation (Table 1). Some interviewed producers mention press reports on ‘two-plotted vegetables’, i.e., farmers who grow vegetables in two distinct plots: only one is safe for home use, and the other is for sale. They claimed that that this practice is no longer found in their production zone. Cooperatives also seem to have effects on the practice transition:

“Because we are a certified cooperative and we sign contracts with canteens […] If any problem of food safety happens, it could harm our reputation” (vegetable producer, #6).

Awareness about pesticides and their consequences and know-how about alternative pest management methods are prerequisites for the acceptance of safety practices. This could result from the training programs on IPM. As a ‘holistic’ and ‘science-based’ approach to combat pests and diseases with minimal application of agrochemical measures (Stenberg 2017), IPM is the dominant crop protection paradigm globally. IPM was introduced in Vietnam in the mid-1990s with the support of international organizations such as FAO, DANIDA, NORAD, and ACIAR (Van Hoi et al. 2013). Training classes have been organized by local PPD and extension agencies for many vegetable farmers. Nearly half of survey respondents stated that they followed IPM protocols, their extensive application shows that the knowledge from this training has also been disseminated among the untrained producers.

The producers also routinize the practice of *using homemade pesticides and physical measures* to prevent insects and weeds (also introduced through IPM training). Smallholders utilize plant-based insect repellents like garlic, chili, lime juice, and other ingredients like alcohol and limestone to produce spraying mixes. Such homemade mixes are sprayed regularly as a complementary pest control method. Physical controls such as simple hand-picking or using insect traps were also adopted by several respondents. This practice requires more care and dedication, as farmers need to regularly visit and observe the fields to detect and physically remove pests and pathogens as soon as possible. This practice is often performed in combination with biopesticide use, yet four respondents have switched to adopt this practice as their sole plant protection method. Minimizing risks for health and safety, especially for self-subsistence purposes, has served as the key meaning for sustaining the practice.

Several interviewees stated *using both biological and chemical pesticides*. Their practice is built around the notion that agrochemicals can be less risky and more effective if used with caution. For example, one interviewee noted:

“Chemical pesticides are only used at the earliest stage, and biopesticides are used afterwards. Because I rotated the crops regularly, I need to use chemicals to remove all the pests and pathogens completely” (vegetable producer, #20).

Balancing cost-effectiveness and safety is the key meaning that guides this practice. Intriguingly, whereas insecticides received much stricter supervision, the use of herbicides seems to face fewer restrictions. Some producers consider herbicides a convenient method of weed control and do not feel the public pressure to use more ‘biological’ alternatives, as in the case of insects and pathogens.

Protocols for *collecting and disposing of waste*, especially pesticide and fertilizer packages, have also been implemented by some producers. In the communes designated as ‘safe production zones’, waste containers are placed in the fields to prevent the packages from being thrown away improperly. Cooperatives seem to have the ability to supervise waste management practices, as one cooperative leader noted that they often ask members to comply with the disposal rule and launch regular field cleaning.

### (Sustainable) intensification practices: the interplay of labor and environment

In addition to crop protection, the smallholders also adopted a variety of intensification practices (Table 1). The bundle ‘(sustainable) intensification practices’ is organized around the notion of maximizing production and productivity for increasing incomes. However, the bundle also involves the idea of improving soil fertility and porosity as well as addressing the lack (or surplus) of rural labor. Hence, small-scale producers attempt to balance the use of more energy-intensive technology with more circular and sustainable practices. The practices are diverse and specific for each producer and area. Traditional knowledge and norms have played a critical role in building the bundle, along with skills and knowledge transferred from IPM trainings and other extension programs.

A prominent practice is the *use of organic fertilizers*, especially chicken manure. Most producers, both in the interviews and the survey, have employed the practice. The utilization of manure for fertilization has a long history, yet it was primarily limited to manure from one’s own farm. Cattle and pigs used to be the main sources of manure before the emergence of chicken farms in Dong Anh district and neighboring regions in the 2000s. Smallholders started to buy manure directly from the farms, and afterwards, traders came directly to each village to sell chicken manure. As the use of organic fertilizer requires storage and transportation facilities and significantly more work and time, the key meaning of practice is to restore and enhance soil health.

“Using chicken manure is more hard work. First, we need vehicles to buy and transport the manure to our home. Then, we have to let it compost until the manure is more porous and ready for use. The weight of manure is much larger, so the workload is higher. If the nitrogen fertilizer is only several kilograms in weight, the amount of organic fertilizer is as large as one hundred kilograms.” (vegetable producer, #38).

The *practice of using synthetic and mineral fertilizers* also remains common. The survey reveals that nearly all producers still rely on mineral fertilizers for ensuring the essential elements for the vegetables, while only six producers primarily used synthetic fertilizers. Synthetic fertilizers are considered fast-acting, thus saving time and work. A quarter of producers use both types of fertilizer with the same ratio. Eleven smallholders reported a transition from using mostly manure to using more synthetic fertilizers, unlike the majority of respondents who followed the opposite direction to use more organic fertilizers.

The outcomes of using organic fertilizers have been reported as positive. The interviewees stated that after using more organic fertilizers, soil fertility and porosity were enhanced. They exhibit a clear understanding of how and why soil conditions are important and why organic fertilization is better for soil health. Some areas, which used to be grazing land or paddy fields, became more productive after being cared for and fertilized with manure. However, there are exceptions; for example, several producers comment that their soil fertility and ecosystems have been declining due to pollution and intensive monoculture.

“The soil used to be great after we used organic fertilizers. But in the last 2–3 years, the soil becomes polluted, the vegetables wither and die. So, I have to do crop rotation, like switching from cabbage to onion and eggplants, to minimize such impacts” (vegetable producer, #37).

*Crop rotation* is nothing new but a traditional practice that has been exercised for generations. However, it has had a different meaning for vegetable producers in recent decades. In the past, farmers rotated between rice and vegetables (e.g., two rice crops and one vegetable season) in a year to ensure food security. Since vegetables are now the main source of livelihood for vegetable producers in the designated zones, the rice-vegetable rotation has been transformed into year-round vegetable production. However, the practice of crop rotation remains persistent; producers switch between different species and varieties of vegetables for climate suitability (“each season, each vegetable”), as the vegetables would be more productive and more pest-resistant. One main reason for crop rotation is to restore soil fertility, which has been harmfully impacted by long-lasting monocropping. Nonetheless, this practice is flexible and may differ per farm.

Smallholders have different approaches to cultivating and preparing the soil. Labor plays a central role in this practice. The reduction in the available labor force (owing to youth migration) contributed to the growing *use of machinery*. Renting machinery for plowing, mowing, and tilling is the more affordable and accessible option for most producers. As the farms are small and not suitable for heavyweight machinery, lightweight equipment is preferred. Nonetheless, even lightweight machinery can affect soil structure and make it more compact during wet weather, so cattle and buffaloes remain used (as shown by a few survey respondents). The producers, however, still widely use manual labor to excerise different manual tasks, as vegetables require ‘more care and skills than other crops’ (vegetable producer, #38). However, the required order of activities has been largely simplified compared to the past. Advanced technologies and know-how help vegetable producers remove unnecessary tasks and improve productivity with less workload.

Finally, practices of *post-harvest field management* are widely adopted. The central idea is to help the soil remove infections and contaminants and revitalize itself. This integrated practice involves several activities. First is the thorough removal of agricultural residues from the fields, which can be collected and composted to recycle nutrients. The practice is important for exterminating habitats for the reproduction of pests and pathogens. Second, they ‘let the soil rest and dry for at least half a month’ to utilize solar heating to eliminate pests and facilitate nutrient recycling and restoration of soil porosity (vegetable producer, #30). Finally, producers pump water and use limestone to remove all the remaining insect eggs and fungi. Although this practice involves much traditional know-how, it has been widely diffused among producers in the last ten years thanks to extension programs. Respondents consider the practice a simple procedure to offset the impacts of intensification and reduce pesticide requirements.

### Post-production and distribution practices

We also explored the changes in the vegetable distribution practices, involving collectors, processors, wholesalers, transporters, retailers, and street vendors. Unlike production practices, the findings show little evidence of any clear transition in distribution practices. However, we identified two key trends. First, the role of cooperatives and cooperative-linked practices have re-emerged. Second, practices of collecting, wholesaling, transporting, and retailing have been diversified under the impacts of urbanization and enhanced means of transport and communication.

### The re-emergence of cooperative practices

Cooperatives used to be the dominant regime actors in Vietnamese food systems in the socialist era. After the late 1980s economic reforms, multiple agricultural production cooperatives were dissolved or transformed into service cooperatives. Nonetheless, after the new Cooperative Laws^2^ and other policies, the government promoted a new model of cooperatives that would be more economically responsible and play a pivotal role in promulgating safety and organic standards and connecting smallholders with the markets (Nguyen-Minh et al. 2023). In communes designated as RAT zones, such restructured cooperatives were established, with around 10–20 households and a democratically elected director board. The director board is responsible for legal and financial management of the cooperatives, while other members can be voluntarily recruited and engaged in other cooperative’s activities.

Our study observed a variety of re-emerging cooperative practices. These practices involve both production (planning, contracting, knowledge, and information sharing, supervising) and post-production (collecting, pre-processing, transporting) activities. For example, at the beginning of each month and each cropping season, the cooperatives *discuss and make plans* about which varieties of vegetables should be grown to meet the orders of their customers. The cooperatives may choose to obtain safety certificates (RAT and VietGAP) and thus become responsible for *supervising* their members to adopt and comply with safety protocols. Another key practice of cooperatives is to organize regular meetings to *exchange information and know-how* (Naziri et al., 2014). Policies and regulations regarding food safety, plant protection, and knowledge about safe practices are disseminated in these meetings.

*Contracting* is one of the key practices involving cooperatives. Certified safe vegetable cooperatives sign contracts with buyers like supermarkets or catering services. Hereby, the board needs entrepreneurial and financial skills to enact and sustain the practice. Following such agreements, cooperatives *collect* vegetable products from their members and exercise practices of *pre-processing and transporting* the products to the customers. This practice, however, faces several risks and constraints. For example, in the case of contracting with supermarkets, payments are often made every 1–2 months. This could generate financial uncertainties for cooperatives composed of mostly smallholder producers with limited savings. Contracting can also be influenced by socioeconomic shocks. During the social distancing period of the COVID-19 pandemic, one cooperative reported that sales were lost due to the closure of catering services for companies, while one cooperative having contracts with a supermarket chain maintained or even increased its profits.

Some risks relate to the potential mismatch between the available quantity and safety of products and those demanded by the markets and customers. Sourcing vegetables from external producers sometimes occurs when demand for certain vegetables exceeds the available quantity on the members’ farms. In some cases, the contracted amount of vegetables was lower than the harvested produce, prompting the members to sell vegetables (at lower prices) through conventional channels like traders or nearby wet markets. Nonetheless, both circumstances entail the risk of mixing certified products with products from unregulated origins. Some respondents see the mixture of certified and non-certified products as the main constraint for them to comply with certifications. The leader of Nam Hong Cooperative stated that his cooperative used to acquire VietGAP certification, but “after two years, we no longer reapply for that. It is too costly to get, but it cannot ensure marketability […] It makes no sense when we paid a lot for the certificate, but we have to sell products to the outside market at the same price as normal vegetables” (In-depth interview, vegetable producer, #11).

### Diversification of wholesaling, transportation, and retailing practices

The distribution practices of small-scale wholesalers and retailers are increasingly professionalized and diversified. This is different from the past practices in the late 1980s and early 1990s, when smallholder producers were also engaged in distribution. While most vegetables grown were for home consumption, the surplus was brought on (motor) bicycles to nearby traditional markets. The vegetable supply chain was short and local with little diversity in vegetable distribution practices.

Since the economic boom in the 1990s, with the expansion of distribution networks and the growing trade between urban and rural areas, more smallholders have become engaged in the vegetable production system but with different roles. The practices of collecting, trading, wholesaling, and retailing started to be routinized by professional practitioners. Infrastructure development and the availability of motorbikes and telephones facilitated their access to different sources as well as to different marketplaces at longer distances. These elements contributed to the diversification of distribution practices, as shown in *Supplemental material*.

There is considerable variation in how small-scale actors buy and sell products in urban, peri-urban, and urban areas. The more the areas are urbanized, the more retailers are sourcing from wholesale markets or other indirect sources. Meanwhile, retailers in urban markets continue to procure most vegetables from home farms and local farms. The rural‒urban difference reflects a divergence in daily lifestyles and routines. Rural and some peri-urban retailers collect products and bring them to nearby wet markets for sale, similar to the traditional practice. For wholesalers and other retailers, the distribution practices mostly involve trading activities at nocturnal wholesale markets, in addition to collecting, gathering, and transporting products and re-selling vegetables at retail markets in the daytime. Overall, the arrangements of the latter practices require more time and work, thus requiring the engagement of more full-time and professional laborers.

*Wholesaling practices* also involve the extensive use of mobile phones. Since wholesalers acquire products from multiple sources, they have to establish their private ‘circle of suppliers’. We also found evidence for digitalization, since wholesalers use messaging applications like Zalo (a Vietnamese mobile app) to support such networking activities. Information about prices, origins, and quality of vegetable products is often exchanged via such apps. New practitioners in wholesaling are often recruited via kinship or close relationships with older practitioners. Necessary entrepreneurial, financial, and communication skills all need to be built up through learning and experience.

“My elder sister also did that job, and she helped me start this business. After some time working with my sister at the wholesale market, I now have my list of suppliers. They are collectors in different regions […] In my Zalo, I often contact them to ask about fresh products. After we negotiate the price, my husband and I drive to the wholesale market in their district to take products. We sort and bring it here to sell to the retailers” (Interview, vegetable wholesaler #291).

For *retailing practices*, new practitioners are recruited more easily. They may be small farmers who just want to sell their surplus vegetables and earn some additional income or full-time sellers who rely on vegetable retailing as their main source of livelihood. They have different motivations and expectations, leading to varied strategies for maintaining, expanding, or subsiding the practices. Motorbikes, as affordable and convenient transportation means, have a critical role in adopting and reproducing the practice. While there is no clear pattern of the changes in retailing practices at the three study sites, there are interesting accounts from individual retailers on how urban development and advances in transportation affected the trajectory of their livelihoods and practices.

“Just seven years ago, I just grew vegetables and sold them at the market near my village. I lived alone with my mother, and I did not have much money to buy stuff. Then, I decided to bring my vegetables to this (urban) market as I can sell them at much higher prices. Every day I wake up early to collect vegetables from my farm. Sometimes, I also take some vegetables from the villagers. I bring all of them– around 70–100 kg in total– using my motorbike. My village is 20 km from here, so it takes about only half an hour in the early morning […] In the afternoon, after finishing, I return home and take care of my farm.” (Interview, vegetable retailer, #287).

*Street vending* is also a retailing practice, yet the difference is that the retailers are often mobile with no fixed location. In urban Hanoi, there are many ‘temporary markets’ organized on streets with no official approval from the government, making the distinction between street vending and other retailing practices less clear. However, our findings suggest that most street vendors are sourcing products from home and local farms, which is different from the reliance of urban market retailers on wholesale markets.

Finally, on safety issues, we found that most interviewed and surveyed distributors expressed concerns about food safety but had little awareness about food safety certificates such as VietGAP. While safety certificates are among the criteria for some surveyed retailers to select vegetables, yet none could explain how those certificates can be adopted. The survey indicates that a more common criteria is to procure from designated and reputed safe production zones (for example, in Dong Anh). Overall, the distributors apply a set of individual skills and knowledge to assess the freshness and safety of vegetables, along with other factors such as locality, seasonality, and personal trust relations with the suppliers, as described in Wertheim-Heck et al. (2014).

## Results: ‘zooming out’ to practice linkages and points of intersection

### Horizontal and vertical linkages

**Fig. 3:**
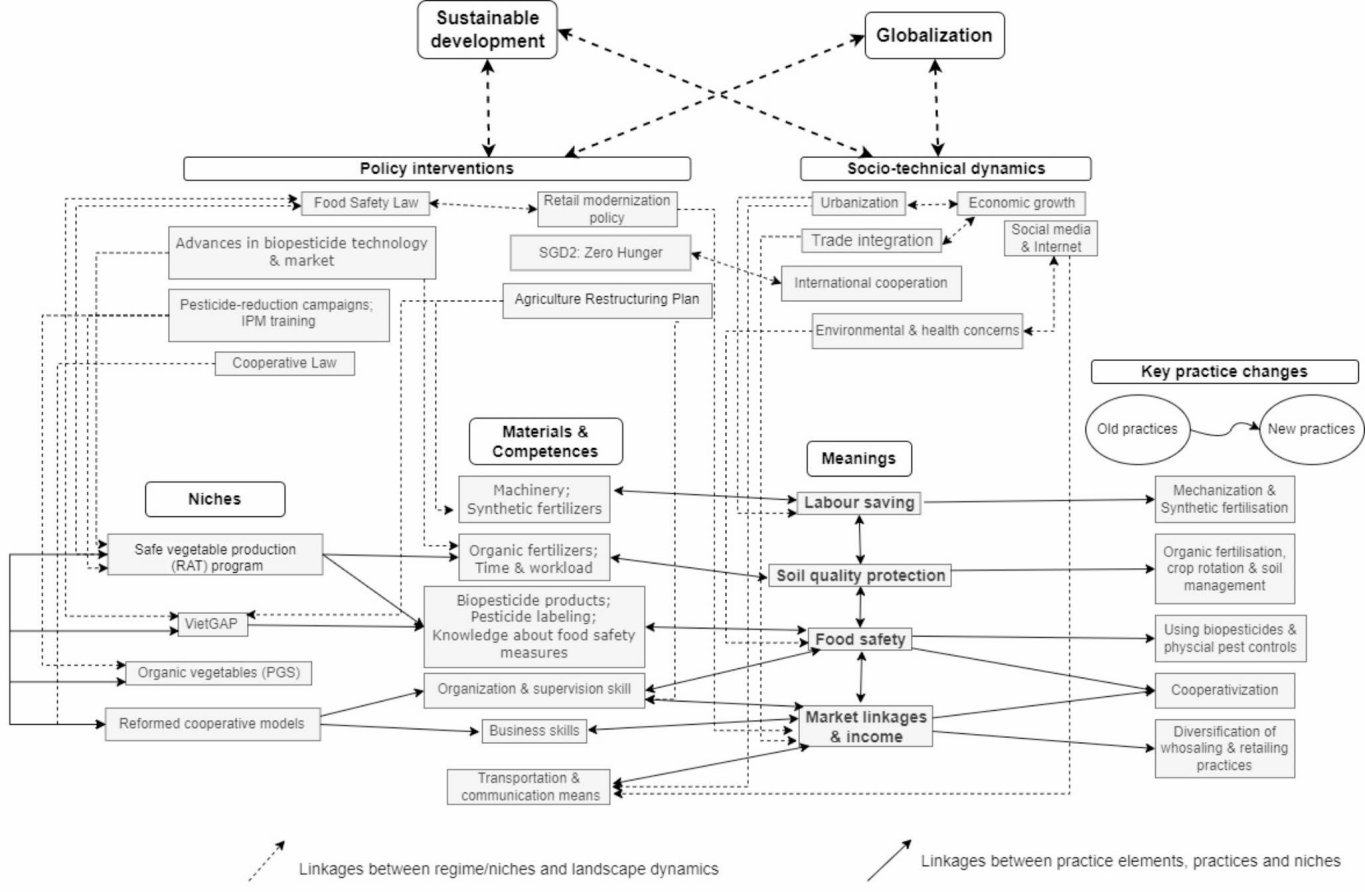
Horizontal links between key meanings, materials, and competencies of small-scale practice dynamics and their vertically linked dynamics. Source: own elaboration

After ‘zooming in’ on the small-scale practice dynamics at the study sites, we explore the horizontal and vertical linkages of those practices at the food system or national level. Figure 3 demonstrates these intra- and cross-level interactions, adapted from the analytical framework (Fig. 1) and the findings from the fieldwork and the desk study.

In Fig. 3, horizontal linkages among elements within practices or between different practices and bundles were identified and mapped. To simplify the combined analysis, we clustered the dynamics in small-scale practices based on the key meanings that guide the directionality of the dynamics. Each key meaning can represent a ‘strategy’ or ‘pathway’ that smallholders embrace to adjust their routinized practices. Those key meanings play a central role in connecting key practice changes with the necessary materials or competences.

Second, the vertical linkages were examined by connecting SPT with MLP analysis of food systems. These strategies and pathways are associated with broader movements and certain interventions across levels. At the national landscape level, two determining socioeconomic processes– urbanization and trade integration– have affected the availability and quality of agricultural inputs like pesticides, machinery, labor, and land. At the regime level, three major policy interventions are prominent: (i) the series of policies and initiatives toward promoting safe production and standardization, (ii) the retail modernization policy that promotes the development of modern retail channels replacing weakly regulated traditional markets, and (iii) the ‘Agricultural Restructuring Plan’ (since 2013) that endorses large-scale farming and subsidizes access to high-tech and sustainable technologies. Such interventions are associated with the establishment of several socio-technical niches, notably the RAT production zones, VietGAP-certified production, and the reformed cooperative models (that might incorporate the former two niches). All of the interventions and processes can be linked with two global attractors: sustainability and industrialization/globalization. These two global principles are continuously introduced and embedded in the international linkages of trade and investment, certification and standards, and the projects of international organization.

### Intersection points

The outcomes from the examination of horizontal and vertical linkages help identify ‘points of interactions’ to denote issues that can block or leverage the diffusion of food safety practices (Fig. 3).

**Fig. 4:**
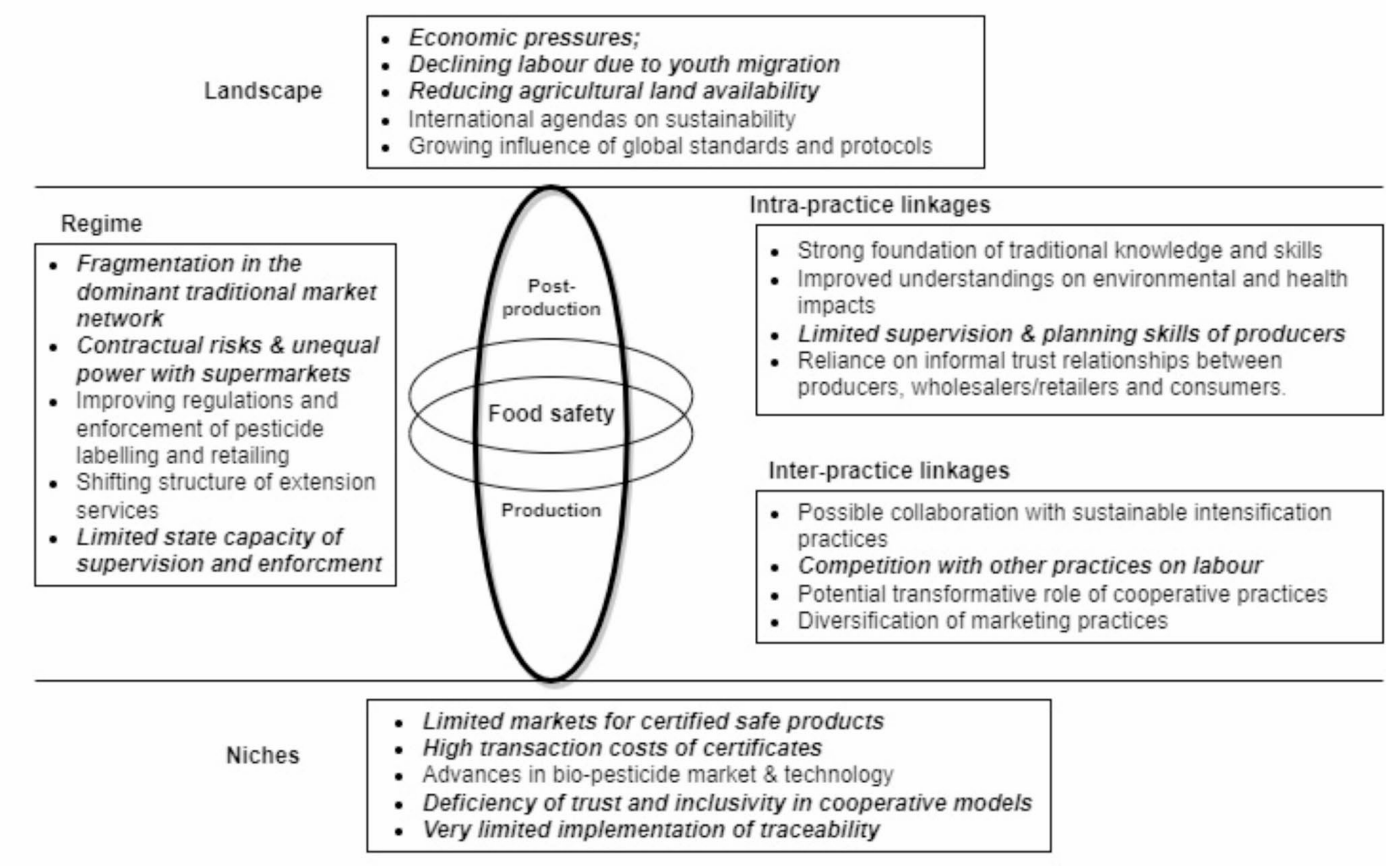
The points of intersection between food safety practices and other dynamics. The italic points indicate the lock-ins that hinder the establishment and upscaling of safe practices Source: own elaboration

First, we specifically explored the interactions of elements within food safety practices. Food safety practices involve myriad elements with various levels of tightness, of which limitations or weaknesses can differently influence the possibility of adopting and reproducing practices. As shown in the previous section (Table 1), enacting safe production practices often requires an understanding of crops, pests, and weather, as well as concerns for human health and the environment. Such competences are shown to be strong for committed producers who have built robust knowledge foundations after intergenerational experiences in vegetable farming. However, small-scale producers generally have less capacity to supervise and plan. Another major limitation lies in the predominance of informal norms and personal trust relationships to verify the idea of ‘safety’, leading to a weak presence of formal institutions like written contracts or certifications (Hoang 2020). Both can affect the possibility of upscaling safe production and diffusing safety standards through supply chains.

Second, food safety practices can also have collaborative or competitive relations with other practices as they might compete over finite material resources or involve significantly different meanings (Watson 2012). For example, the uses of *biopesticides* and *chemical pesticides* compete over money and farm habitats as well as in the perceptions of safety and responsibility, while the use of *physical crop protection* might compete with intensification measures over the requirement of labor and time. Collaboration happens when a practice helps to produce elements that are necessary for sustaining others. Safety practices could collaborate with more sustainable intensification practices, especially *crop rotation* and *post-harvest soil management* which have flexible meanings and implementation methods. Both practices can be exercised seasonally or yearly to remove remaining pests and reduce pesticide requirements, along with the other purposes of recycling nutrients and restoring long-term soil fertility. Another example is the role of emerging *cooperative practices*, which integrate information sharing, supervision, planning, and marketing practices to ‘filter’ certain practices of crop protection and variety selection. The bundle hence has the potential to transform farming practices by creating linkages with new knowledge and meanings on food safety and sustainability and building relations with new networks involving safe food buyers, intermediaries, and research and extension institutions.

Finally, using the combined findings from the desk study and the fieldwork, we scrutinized and highlighted specific dynamics within landscape, regime, and niche levels that potentially influence the performance of food safety practices. Building on the above outline of horizontal and vertical linkages, we identified multiple intersection points between the reproduction of safety practices and multi-level dynamics. These points are summarized in Fig. 4, inspired by the works of Gazull et al. (2019) and Hargreaves et al. (2013). The figure presents different factors that hinder the diffusion of food safety protocols, from the internal weaknesses of practitioners to the competition and mismatches with other practices, from the external risks and blockages within the regimes to the overwhelming landscape pressures. We will further discuss this aspect in the next section.

## Discussion

### The use of social practice and multi-level analysis

This study has examined the dynamic practices of small-scale actors in the Vietnamese vegetable sector, to unfold how and why smallholder practices have changed over the last 20 years, and what can explain the success and limitations of adopting safe food practices. We hereby drew on the SPT and MLP theoretical frameworks. The findings reveal several key patterns of small-scale practice changes, especially the switch to using biopesticides as well as the growing adoption of sustainable intensification practices (organic fertilization, soil management), although uses of synthetic fertilizers and machines remain common. Furthermore, the results provide a detailed description of the different materials, competencies, and meanings involved in different (bundles of) practices. Materials like finance, time, labour, agri-inputs, soil, machines, and weather, along with multiple competencies and meanings are combined in normalizing and routinizing the practices.

This research goes beyond the conventional field of consumer practices (Svennevik 2022) and adds to the small yet growing number of studies that explore the social practices of food provisioning (Adeosun et al. 2022; Swagemakers et al. 2012; Ulug et al. 2021). However, we also recognized some major difficulties when adopting SPT and MLP to analyze smallholder provisioning practices. The study had to address multiple timespans of provisioning practices, not just daily routines but also seasonal and yearly habitual activities. These practices are also situated in compounded networks involving myriad actors, including human and non-human agents, intersected with a wide range of institutional and market arrangements. Hence, the analysis of provisioning practices could not be limited to the conventional ‘social practice model’ (practices as intermediating between lifestyles and systems of provision), as in most studies of consumption practices (Spaargaren 2003).

Therefore, our study also adopted concepts from transition theory to expand the analytical lens and identify the intersections of smallholder practices with niche, regime, and landscape dynamics. The results show how SPT and MLP are complementary in understanding the complex arrangements of practices and institutions. Applying both approaches for qualitative analysis allows for understanding practice-as-entity with particular local enactments.

However, we acknowledge that the findings are mainly applicable for the studied communes and districts in Hanoi and Hung Yen. With a limited sample size and non-probability sampling methods, the study cannot generalise and evaluate the practice-as-performance of Vietnamese or even RRD smallholder vegetables. This invites future research combining our exploratory, qualitative analysis of social practice and multi-level dynamics with robust quantitative studies to assess smallholder practices, drivers, and performance outcomes thoroughly.

### Stability and changes in smallholder practices: a food safety transition?

This study emphasizes the combination of stability, change, and diversity in small-scale vegetable supply practices. Stability in smallholder practices, like consumer practices, is reinforced by the continuous reproduction of practice elements. Our findings demonstrate that for all producers, their vegetable production practices maintain some essential characteristics of small-scale, artisanal farming. Smallholders are limited in their land, capital, and labor endowments and are inclined to adopt routines that resemble the old, traditional patterns. In that view, using organic fertilizer, reducing tillage, or adopting crop rotation and post-harvest soil management can be considered the re-enactment of old practices with newly adjusted meanings. Even in the case of using biopesticides, the purchasing and spraying routines do not fundamentally diverge from the conventional ones, while the main differences are the recognition of biopesticide labeling and the active role of pesticide dealers.

Although we recognized the inclination of smallholder practices to stabilize, there are also different accounts of novelty and change in the case study. Many respondents, especially those associated with cooperatives, reported a switch to adopt safety protocols (guaranteeing PHIs and using biopesticides) and organic fertilizers, whereas others maintained the use of chemical pesticides or synthetic fertilizers. The differences in the acceptance and pace of diffusion among smallholders indicate that the smallholder community is not homogeneous or static but heterogeneous and dynamic (HLPE 2013; Kaiser and Burger 2022). This diversity can be attributed not only to the multiplicity of geographic, socio-economic, and ecological backgrounds (Iles et al. 2020) but also to the differences in interest and motivation among practitioners (Warde 2005). Respondents have rationally explained why they perform certain crop protection or intensification practices, citing one among three key meanings of “food safety”, “labor-saving” and “soil quality protection” as the core purposes, while not excluding common purposes like “maintaining farm business” or “earning income”. This echoes the notion of Huttunen and Oosterveer (2017) that there should be ‘hierarchically organized purposes’ in prioritizing the adoption of practices, signifying the critical importance of agency in shaping practice transitions.

The study outlined several technological and socio-institutional developments that impacted the transition of smallholder practices. Policy interventions like IPM training and regulations on pesticides, along with the enhanced access to advanced technologies and the development of other regimes (e.g., the livestock sector), offered new materials and competencies for enacting new practices. Urbanization and modernization are also major determinants of novelties: urban developments encroached and put pressure on farm activities, and small-scale distributors adapt to changing urban and market infrastructures in diverse ways. Additionally, ideas from the safety and sustainability discourses affected the shifting of crop protection and intensification practices.

Our study suggests a certain degree of success of the long-term interventions promoting IPM and safer pesticide usage (Hoi et al. 2016) that led to the diffusion of biopesticides in Dong Anh. However, validating this requires more empirical data, considering that biopesticide products constitute only a small (but growing) share of the global pesticide market (Damalas and Koutroubas 2018). Meanwhile, there were no clear normative changes in distribution practices, except for a small group involved in cooperatives. The diversification of distribution practices was not linked with ‘food safety meanings’ but only to maintain their business and earn an income by adapting to urbanizing contexts. The limited motivation among distributors to change also indicates lock-in effects in the dominant regime (Fig. 4): fragmented traditional markets with inadequate food surveillance and low consumer trust and acceptance of certified safe products (Nguyen-Viet et al. 2017). Even for cooperative practices, the possibility of upscaling is constrained by unequal negotiating power and difficulties in recruiting new members (who must share existing visions and comply with cooperatives’ supervision). These lock-ins could hinder the diffusion of safety practices beyond safe production zones and cooperatives.

Our findings enrich the ongoing debate about food safety policy in Vietnam and other LMICs. The neoliberal policy approach that emphasizes “supermarketization” and global standards is emphasized as limited in flexibility and inclusiveness (Hoang 2020; Wertheim-Heck et al. 2015), but there are also few results in generating practice changes within traditional distribution networks. It highlights the need for more innovations and interventions that simultaneously target various components of vegetable systems, rather than just production, to enact its reconfiguration toward food safety (Bui 2021).

### Future ‘pathways’ of smallholders: prospects for policies

Our last discussion emphasizes that policy interventions should acknowledge the multiplicity in smallholders’ interests, which influence their strategies in adjusting practices. While our study did not attempt to provide a typology of smallholder pathways like some previous studies (Huttunen 2019; Kaiser and Burger 2022), we suggest that the three key meanings of ‘food safety’, ‘saving labor’ and ‘soil environment protection’ interact to shape the transitions of smallholder production practices. Among these three meanings, labor has been a constraining factor that influences smallholder decisions to either sustain more labor-intensive practices (using organic fertilizers or physical pest controls) or to adopt labor-reducing technologies. Considering the short cycle of vegetable crops (i.e., requiring more workload to care) and the typically small landholdings (Huong et al. 2013b), interventions for promoting safe or sustainable vegetable practices in Vietnam should prioritize considering the availability of labor on farms, which seems to decline over time due to youth migration.

Soil fertility is traditionally a vital concern for smallholders who want to sustain long-term productivity. This explains why multiple smallholders adopt organic fertilizers and other soil cultivation practices despite fewer public interventions to protect the soil environment. However, the adoption of such practices, as our case shows, is mainly organized around the idea of securing long-term soil productivity to maintain farm business. This implies that care about the environment might not be the priority of smallholders but that this is embedded in the self-sustaining meaning shaped by path dependency and formative experiences (Hedberg and Zimmerer 2020). Hence, future initiatives should seek to engage the current soil management practices with broader meanings, especially for promoting coordinated actions to cope with ongoing threats to land availability and the soil environment, such as pollution and land grabbing due to industrial and urban development.

Last, food safety is the most strictly steered pathway among the three meanings, with many interventions and niches promoting safe production practices. Such initiatives, along with the public discourse on food safety, are proven to be partially successful in pulling smallholders to adopt safer practices. Smallholders found more institutional support to follow the food safety pathway rather than other goals of soil and environmental protection (Baur 2020) and could more easily associate their practice changes with the grand narratives of sustainability and globalization by following the global protocols of IPM and GAP. However, as discussed in the previous section, such transitions in production practices are misaligned with the inertia in distribution practices and institutions.

## Conclusion

Our study explored the transitioning practices of Vietnamese vegetable smallholders during the last 20 years. We found that smallholder practices tend to stabilize due to path dependency and limited capital availability but also identified different accounts of changes and opportunities for innovation. It is intriguing to observe that in our case, smallholder producers were generally not resistant to adopting safety practices, yet it was the inertia in distribution networks that hampered the possibility of upscaling them and transforming the whole supply chain. Food safety, labor, and soil fertility together constitute the three key meanings that constrain or steer pathways of smallholder practices. Their interplay under the landscape pressures of globalization/industrialization and sustainable development has significant impacts on smallholder practices and decisions, either to fulfill a single meaning or to find a balance among the three of them, to ‘move up’ and upscale good practices, or to ‘move out’ to find alternative incomes (Fan and Rue 2020). This study calls for future research and policies that acknowledge the various interactions of smallholder practices and their key meanings and recognize the crucial role that smallholders play in delivering food safety outcomes and other sustainable goals in the developing world.

## Electronic Supplementary Material

Below is the link to the electronic supplementary material.

Supplementary Material 1 (DOCX 19 KB)

## Footnotes

1 Although smallholders mostly refers only to producers with small-scale farm size (usually less than 2 ha of landholding), we used ‘small-scale producers and distributors’ and ‘smallholders’ interchangeably in this paper.

2 Cooperative Laws were issued in 1996 and revised in 2003, 2012 and 2023. The latest Cooperative Law (17/2023/QH15) was promulgated on 20 June, 2023 by The Assembly of Vietnam.
