# Supplementary material for: Transitioning practices of vegetable small-scale actors in Vietnam: an interplay of food safety, labor demand, and soil environment
Source: Agric Human Values. 2024 Oct 23;42(2):825–43. doi: 10.1007/s10460-024-10636-6 (PMC12098484; doi:10.1007/s10460-024-10636-6)
Supplement: Supplementary file 1 — Supplementary Material 1 (DOCX 19 KB) [file 10460_2024_10636_MOESM1_ESM.docx]

**Supplemental materials**

# Descriptive characteristics of survey respondents

| **Group** | **Sex (% of female)** | **Age (yrs)** | **Household size (number of people)** | **Income level*** | **Educational level**** | **Livelihood diversity**  ***** (number)** | **Farm size (m^2^)** | **Number of farm plots** |
| --- | --- | --- | --- | --- | --- | --- | --- | --- |
| Vegetable producers (n=56) | 89.9% | 53.3 ± 9.8 | 4.3 ± 1.6 | 3.7 ± 1.4 | 2.3 ± 0.8 | 2.3 ± 1.1 | 2155 ± 1141 | 3.9 ± 2.8 |
| Vegetable distributors (n=67) | 95.8% | 47.3 ± 9.7 | 4.5 ± 1.7 | 3.9 ± 1.2 | 2.3 ± 0.7 | 2.0 ± 1.0 | NA | NA |
| Note: * Levels of monthly household income per capita (1-5): <1.5, 1.4-2.4, 2.4-3.4, 3.4-4.9, >4.9 million Viet Nam Dong (quintiles based on Viet Nam Household Living Standards Survey 2018)  ** Educational levels (1-6): primary school, secondary school, high school, public college/professional high school, bachelor’s degree, post-graduate.  *** Livelihood diversity: the total number of livelihood activities of all members currently living in the household. | | | | | | | | |

# List of key informant interviews

| **Code** | **Organization and position** | **Date interviewed** |
| --- | --- | --- |
| K1 | Market Management Board – Head | 2020-12-02 |
| K2 | Department of Economy - Staff | 2020-12-03 |
| K3 | Director of Dong Anh Plant Protection Agency - Director | 2020-12-15 |
| K4 | Dong Anh Plant Protection Agency – Vice Director | 2020-12-15 |
| K5 | Tang My (Nam Hong) Safe Vegetable Cooperative – Director | 2020-11-17 |
| K6 | Son Du (Nguyen Khe) Cooperative – Director | 2020-11-17 |
| K7 | Co Loa Agricultural Service Cooperative - Director | 2020-12-03 |
| K8 | Cau Giay Management Board – Director | 2020-12-12 |
| K9 | Minh Khai Market Management Board – Manager | 2021-01-07 |

# Characteristics of wholesaling and retailing practices

| Distribution practices | Daily trading quantity | Buying from | Selling to | Required means |
| --- | --- | --- | --- | --- |
| Wholesaling (n = 22) | 50-5000 kg/day | Home farms (9); Local farms and cooperatives (6); Other wholesalers (4); Relatives and friends (1); Reputed farms and cooperatives in the province (2); Traders from other provinces and China (2) | Retailers; catering services; restaurants; supermarkets; individual consumers | Automobiles & motorbikes; telephone with message app |
| Retailing at rural markets (n = 11) | 10-200 kg/day | Home farms (6); Local farms (4); Wholesale market (1); Relatives (1) | Individual consumers; restaurants | Bicycles, motorbikes |
| Retailing at peri-urban wet markets (n = 17) | 10-300 kg/day | Wholesale market (8); Local farms (7); Home farms (5) | Individual consumers; restaurants; relatives; traders; | Motorbikes |
| Retailing at urban wet markets (n = 22) | 10-200 kg/day | Wholesale markets (17); Local farms (5); Home farms (4); | Individual consumers; restaurants | Motorbikes |
| Street vending in urban areas (n = 10) | 20-100 kg/day | Home farm (7); Local farms (2); Wholesale market (3) | Individual consumers; restaurants; relatives; | Bicycles, motorbikes |
